# Carpal Tunnel Syndrome Resulting From Persistent Median Artery and Bifid Median Nerve: The Critical Role of Ultrasonography

**DOI:** 10.7759/cureus.54551

**Published:** 2024-02-20

**Authors:** Mustafa Turgut Yildizgoren, Cahit Ucar

**Affiliations:** 1 Department of Physical Medicine and Rehabilitation, Konya City Hospital, Konya, TUR; 2 Department of Internal Medicine, Necmettin Erbakan University, Medical School, Konya, TUR

**Keywords:** carpal tunnel syndrome, persistent median artery, bifid median nerve, treatment choices, pain

## Abstract

Here, we present a 37-year-old right-handed female patient who presented with increasing paresthesia, tingling, and numbness in the radial palm and lateral three fingers of her right hand for six months. Because of the intense wrist discomfort and unilateral involvement, ultrasonography was used to evaluate the patient in addition to a nerve conduction study to exclude secondary causes of carpal tunnel syndrome.

## Introduction

The most common peripheral entrapment neuropathy, carpal tunnel syndrome (CTS), is more common in women. The pathophysiology that underlies them is not well understood. Nonetheless, it is stated that nerve edema, decreased blood flow, and congestion of the endoneural and epineural veins play a part [1]. The patient's medical history, physical examination results, diagnostic testing, and electrophysiological investigations are used to diagnose CTS. In patients who refuse invasive nerve assessment procedures, imaging techniques are frequently required to identify issues and structural abnormalities, such as cysts or tumors, and validate the clinical diagnosis [2]. Magnetic resonance imaging, or ultrasonography, is recommended for this reason. An ultrasound examination can also show structural changes (bifid median nerve, persisting median artery, etc.), flexor tenosynovitis, displaced bone, ganglia, or other lesions in the tunnel that occupy space as potential causes of chronic pain syndrome. For the sonographic diagnosis, both longitudinal and transverse pictures are required [3-5]. The severity of the condition, the length of the symptoms, and the patient's preferences all influence the available CTS treatments. Treatments for CTS vary and fall into two categories: conservative and surgical techniques [6-8]. This is a rare instance of chronic median artery linkage with the bifid median nerve, a secondary cause of CTS. Furthermore, the use of ultrasonography in diagnosis and therapy planning is highlighted.

## Case presentation

**Figure 1:**
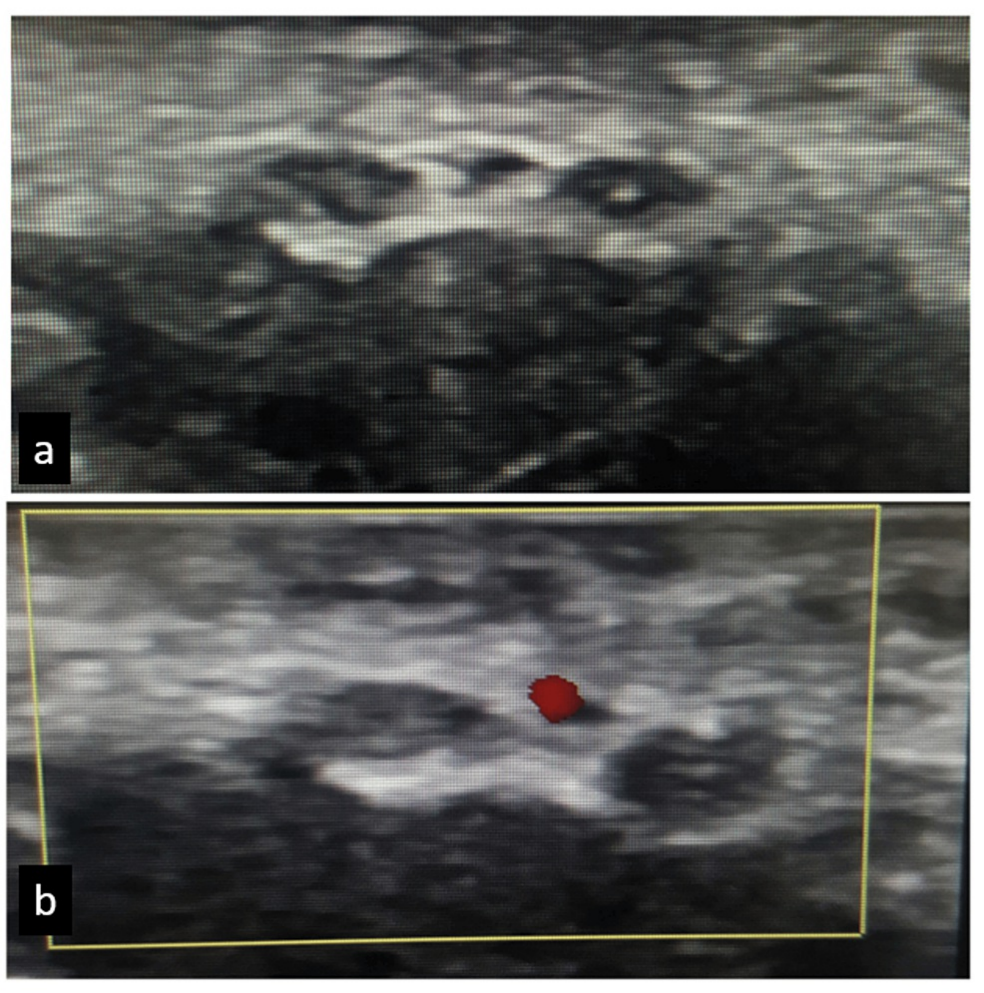
(a) B-mode and (b) color Doppler images of transverse ultrasonography of the right wrist showing the bifid median nerve and the persistent median artery

A 37-year-old female patient, a housewife, who was right-handed, arrived with a six-month history of gradually increasing paresthesias, tingling, and numbness in her right hand's radial palm and lateral three digits. She spends a lot of time doing housework during the day. The patient stated that she had severe pain and numbness at night (visual analogue scale night: 6) and that she had no pain during the day. Her arms did not exhibit any radicular symptoms or neck discomfort. She had no known systemic disease in her history other than hyperlipidemia. Tendon reflexes were symmetrical and normal. There was a noticeable reduction in feeling for light touch and pinpricks in the right hand's lateral three fingers. A Tinel sign was not elicited. On the right wrist, however, a Phalen test result was positive. In the nerve conduction test, she was diagnosed with moderate CTS due to the slowing speeds of sensory conduction, associated with an increased distal motor delay of the median nerve. Due to intense wrist discomfort and unilateral involvement, ultrasonography was used to assess the patient and rule out any secondary causes of CTS. A linear Clarius ultrasound scanner (L7, Clarius, Burnaby, BC, Canada; frequency range: 4-13 MHz, depth: 1-7 cm) was used for imaging. The median nerve was shown to be bifid under the flexor retinaculum on ultrasonography, and the median artery was still present in the center. In addition, the median nerve's cross-sectional area was measured at 17 mm^2^, and it was thickened at the level of the pisiform bone in the carpal tunnel. Figure 1 shows the B-mode transverse ultrasonographic image (a) and color Doppler mode (b).

**Figure 2:**
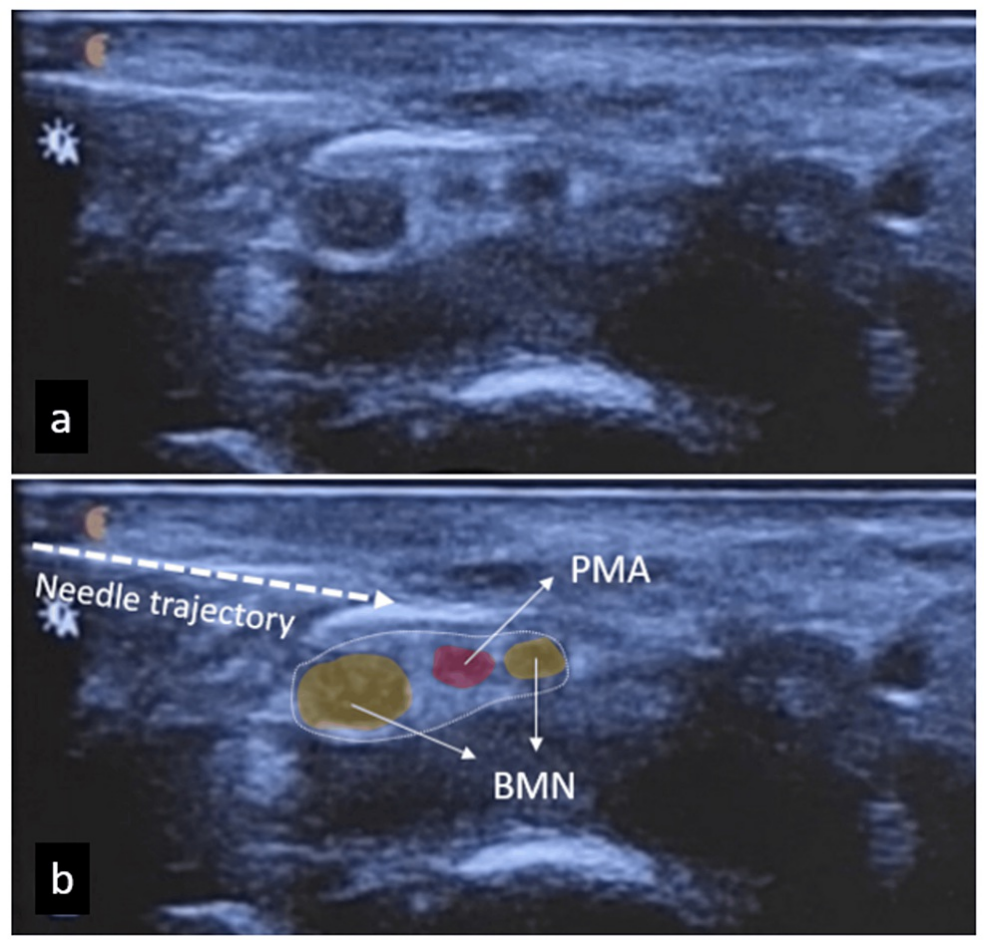
(a) Transverse ultrasonography of the right wrist showing the bifid median nerve and the persistent median artery. The image also shows ultrasound-guided injection and needle trajectory. The bottom image (b) is colored to distinguish the relevant structures PMA: persistent median artery (red), BMN: bifid median nerve (yellow), needle (dashed line)

A combination of glucocorticoid (1 ml, 40 mg methylprednisolone) and local anesthetic (1 ml, 2% lidocaine) was administered from lateral to medial with an in-plane approach under ultrasound guidance to treat numbness and discomfort in the right hand (Figure 2). The patient was recommended a nighttime wrist splint to relieve her symptoms. It was recommended to use vitamin B complexes and painkillers if necessary. She was called for an examination again a month later. During the follow-up, it was learned that her complaints were not relieved despite her regular use of medications and splints. Stating that his symptoms were gradually worsening, the patient was referred to surgery for endoscopic release.

## Discussion

The bifid median nerve and persistent median artery association are very rare anomalies seen in the population. Although the reported prevalence of bifid median nerves has ranged from 2% to 26% per wrist, the prevalence of bifid median nerves and persistent median arteries in the general population is delineated poorly [8]. Treatment options for CTS depend on the severity of the disease, duration of symptoms, and patient preferences. CTS has different treatments, which can be classified as conservative or surgical methods. Conservative treatments include activity modification, hand-wrist splints, local corticosteroid injections, oral medications (nonsteroidal anti-inflammatory drugs, painkillers), vitamin B6, electrotherapy (transcutaneous electrical nerve stimulation, therapeutic ultrasound, laser therapy), stretching, strengthening, nerve gliding exercises, and lifestyle changes [9]. In most cases, it is necessary to approach patients with conservative treatment. Surgical intervention should be considered for individuals with severe disease or who do not respond to conservative methods. Wrist splints are preferred for initial treatment and are usually worn overnight to maintain a neutral wrist position. Oral medication is also added. Patients should have a follow-up evaluation within one to two months. If symptoms improve, it is recommended to continue splinting. However, if improvement cannot be achieved, the option of combining splinting with other therapeutic approaches should be considered. Although electrotherapy and superficial or deep hot applications are beneficial for the patient in the treatment of primary CTS, adequate results cannot be obtained with conservative treatments for secondary CTS.

Imaging methods are often necessary to reveal problems and structural abnormalities, such as cysts or tumors, and to confirm the clinical diagnosis in patients who do not accept invasive nerve evaluation studies. For this purpose, ultrasound or magnetic resonance imaging is preferred [10,11]. Ultrasound examination can also depict possible causes of CTS, e.g., anatomical variations (bifid median nerve, persistent median artery, etc.), flexor tenosynovitis, displaced bone, ganglia, or other space-occupying lesions in the tunnel. Transverse and longitudinal images are both necessary for the sonographic diagnosis. Typically, the nerve is observed to be thinner at the site of entrapment and enlarged just proximal to it. In the diagnosis of CTS, combining electrophysiological studies and imaging studies is very useful for the physician in guiding the treatment plan.

## Conclusions

The bifid median nerve and persistent median artery association are extremely rare in the population. Such rare anomalies may cause undesirable nerve injuries during surgery. We think that knowing what the surgeon will encounter during surgery will increase both treatment selection and treatment success. Therefore, ultrasonographic evaluation should be included in the diagnostic protocol, and the surgeon should be informed in advance about the current situation.
